# Obesity education in the family medicine clerkship: a US and Canadian survey of clerkship directors’ beliefs, barriers, and curriculum content

**DOI:** 10.1186/s12909-019-1614-y

**Published:** 2019-05-27

**Authors:** Harland Holman, Sumi Dey, Ian Drobish, Leora Aquino, Alan T. Davis, Tracy J. Koehler, Rebecca Malouin

**Affiliations:** 1 Michigan State University College of Human Medicine, 909 Fee Road, B201, East Lansing, MI 48824 USA; 2 Spectrum Health Family Medicine Department, 25 Michigan Ave, Suite 5100, Grand Rapids, MI 49503 USA; 3 Spectrum Health, Office of Medical Education, 945 Ottawa Ave NW, Grand Rapids, MI 49503 USA

**Keywords:** Obesity, Medical education, Primary care, Obesity bias, Attitudes, Family medicine

## Abstract

**Background:**

Despite concerns regarding the increasing obesity epidemic, little is known regarding obesity curricula in medical education. Medical school family medicine clerkships address common primary care topics during clinical training. However, studies have shown that many family physicians feel unprepared at addressing obesity. The purpose of this study was to evaluate factors related to obesity education provided during family medicine clerkships as well as identify future plans regarding obesity education.

**Methods:**

Data were collected through the 2017 Educational Research Alliance (CERA) survey of Family Medicine Clerkship Directors (CDs) in the United States and Canada. Survey items included the level of importance of obesity education, teaching methods, barriers to teaching, and obesity related topics taught during the clerkship. Survey data were summarized and analyzed.

**Results:**

The survey response rate was 71.2%. The most frequent barrier to teaching obesity related topics was time constraints (89%). The most commonly taught topics were co-morbid conditions (82.1%), diet (76.9%), and exercise (76.9%). The least commonly taught topics were addressed less than 30% of the time, and included cultural aspects, obesity bias, medications than can cause weight gain, medications to treat obesity, and bariatric surgery. Over half of CDs (59%) are not planning to change existing curriculum, with 39% planning to add to the current curriculum. The CDs’ perceptions of the importance of obesity education were significantly associated with the number of topics covered during clerkship (*p* <  0.001). No relationship was found between clerkship duration and the number of obesity topics taught.

**Conclusion:**

The majority of clerkship directors are planning no changes to their existing curricula which consist of three common topics: obesity related co-morbid conditions, diet, and exercise. While time was the largest self-rated barrier in teaching obesity related topics, clerkship duration didn’t impact the number of topics taught. However, the relative amount of importance placed by CDs upon obesity education was significantly associated with the number of topics covered during clerkship.

## Background

According to a recent study, the National Center for Health Statistics (NCHS) at the Center for Disease Control estimated that from 1999 to 2014, obesity prevalence increased from 30.5 to 37.7% in adults, and 13.9 to 17.2% in youth [1]. Data from a 2015–2016 survey performed by the National Center for Health Statistics at the CDC reported an overall prevalence of obesity in adults at 39.8% [1]. At the current pace, all American adults are expected to be overweight or obese by 2048 [2]. The implications of this are far-ranging, as it is well known that obesity is associated with chronic medical conditions (e.g., diabetes mellitus (DM), coronary artery disease (CAD), hypertension (HTN), chronic mental illness). A recent study estimated obesity-induced heath care costs to approach $210 billion per year [3], with the indirect costs on society being much higher than the measurable healthcare costs [4]. Moreover, obesity is associated with reduced work productivity [5] and increased work absenteeism [6]—with a concomitant cost of $4.3 billion annually [6].

Despite obesity having a substantial effect on patient care in all disciplines in medicine, including primary care, surgery, and psychiatry, it seems physicians’ and medical students’ education about obesity is often limited and inadequate [7, 8], and historically has been received from sources outside of their formal medical school education [9]. These educational gaps may leave physicians underprepared to address the needs of this substantial and growing portion of their patient population.

Nutrition is a key aspect of the obesity epidemic. Recent studies have found that medical students are are receptive to learning about nutrition and view education on adopting health promoting eating patterns as a vital part of healthcare, but feel ill-equipped to provide this care [10].

One study reported that during 2008–2009 only 27% of medical schools followed the National Academy of Science curriculum minimum of 25 required hours of education regarding nutrition [7]. The inadequate amount of dedicated time to nutrition education is concerning, when considered with the results of a recent survey where only 14% of residents felt physicians were adequately trained to provide counseling on nutrition [11]. Similarly, 72% of surveyed primary care physicians felt they had inadequate training on obesity management during medical school and did not consider themselves to be effective in counseling patients on obesity, though 73.5% believed it was their job to do so [8]. Furthermore, a study performed in 2013 demonstrated a decline in physician-led weight counseling while obesity rates have continued to increase [12].

Primary care physicians are often involved in medical education and influence the content of the medical school curriculum. For example, the family medicine clerkship directors (CDs) are responsible for determining the various educational topics included within the clerkship. However, there is a paucity of data regarding the quantity or content of both obesity education and the various factors that influence the decision to include this content within family medicine clerkships. A recent study from Norway found that medical students began with insufficient knowledge regarding obesity management but had improvement throughout their medical education [13].

We hypothesized that:1) The family medicine CDs’ population of patients who are obese in their practice is the main demographic predictor of the number of obesity-related topics 2) Most family medicine clerkships have a traditional curriculum related to obesity (objectives, oral, and written exam materials), but few have applied methods such as simulated patients and cooking classes.

3) Time is the largest barrier to teaching about obesity during the clerkship.

4) Most CDs plan to increase the obesity-related curriculum in the next three years around obesity given the national growing epidemic.

The purpose of this study was to explore factors related to obesity related education provided during family medicine clerkships, as well as identify future plans regarding obesity education. Determining these factors may help inform future decisions around obesity education in family medicine clerkships and medical school curricula in general.

## Methods

### Study design

This cross-sectional survey was distributed annually to the institutional representatives of qualifying medical schools. The institutional representative is the CD at the main campus of the school or their designee. The survey contained 45 questions with 18 questions regarding characteristics of the CD, medical school, and general questions of the clerkship (duration, setting, etc). These questions were reviewed with CAFM. There were 6 obesity related questions. Most questions gave multiple choice responses and allowed CDs to make more than one choice. An example question is, “Which of the following obesity topics are taught during your family medicine clerkship? Choose all that apply.” Questions also were asked about barriers, future plans, and methods of teaching. Attitudes about the importance of obesity were assessed using a 5 point Likert scale. We believe this and other questions have not been asked before in the literature. The demographic questions had been used for several years and published prior. Approved projects were assigned a CERA Research Mentor to help refine questions. The final draft of survey questions was then modified following pilot-testing.

Subjects and survey procedure: In 2017, there were 125 U.S. and 16 Canadian unique individuals identified as family medicine educators directing a family medicine or primary care clerkship. CAFM members were invited to propose survey questions for inclusion into the CERA survey. The survey was distributed via email invitation to 125 U.S. and 16 Canadian family medicine CDs between June and August, 2017 There were two US emails that were undeliverable, reducing the final sample size to 139. Invitations to participate in the study included a personalized greeting and a letter signed by the presidents of each of the four sponsoring organizations with a link to the survey, which was conducted through the online program SurveyMonkey® (. During the course of the survey, 17 changes to CDs were identified, 14 through contact with the survey director and 3 in the survey. All new CDs were then invited to participate in the survey. Non-respondents received weekly requests to complete the survey via SurveyMonkey® (San Mateo, CA). Additionally, they were also contacted through personal email to verify their status as CDs, to check accuracy of email addresses, and to encourage participation. The study was approved by the American Academy of Family Physicians Institutional Review Board.

### Data collection

Data were gathered and analyzed as part of the 2017 Council of Academic Family Medicine’s (CAFM) Educational Research Alliance (CERA) survey of Family Medicine Clerkship Directors (CDs). CAFM is a joint initiative of four major academic family medicine organizations, including Society of Teachers of Family Medicine, North American Primary Care Research Group, Association of Departments of Family Medicine and Association of Family Medicine Residency Directors. The cross-sectional survey is distributed annually to the institutional representatives of qualifying medical schools. The institutional representative is the CD at the main campus of the school or their designee. Qualifying medical schools are accredited by the Liaison Committee on Medical Education (LCME) or CACMS (Committee on Accreditation of Canadian Medical Schools) and are located within the United States of America and Canada. To qualify, the school must have students who complete a family medicine clerkship or a primary care clerkship that has a required family medicine component with a family medicine educator responsible for that component.

#### Survey questionnaire

CDs were asked basic demographic questions, including the number of the years they have served as CD, gender, ethnicity, race, degree, year of graduation, and design/length of the family medicine clerkship. CDs were then queried about the obesity topics taught during the family medicine clerkship, the methods used to teach those topics, the barriers to obesity education, their future plans related to obesity curriculum, the percentage of their own patients that are obese, and the importance of obesity education.

#### Analyses

Summary statistics were calculated. Numericaldata are expressed as the mean + SD, while nominal data are expressed as a percentage. Comparisons between groups for quantitative variables were performed using the one way ANOVA using Tukey’s post hoc analysis to assess significant differences. Nominal variables were evaluated using the Fisher’s Exact test. Linear regression was performed, using number of obesity topics taught as the dependent variable and clerkship duration (defined as < 6 weeks or > 6 weeks) and importance of teaching obesity related topics classified as not at all important, slightly important (SI), moderately important (MI), and extremely important (EI) as independent variables. Significance was assessed at *p* <  0.05. Analyses were performed using Stata v.15.0 (StataCorp, College Station, TX).

## Results

The overall response rate to the survey was 71.2%, with 99 of 139 CDs responding. However, not all respondents answered every question. The majority of the CDs who responded to the importance of teaching obesity related topics question selected the VI option (42/95, 44.2%), followed by MI (28/95, 29.5%), EI (18/95, 18.9%) and SI (7/95, 7.4%). No CDs selected the “not at all important” designation. Of the CDs that see patients, most respondents estimated the percentage of their patient population who are obese in the 26–50% (36/94, 37.9%) or 51–75% (48/94, 58.5%). There were no significant differences among the importance of obesity education categories with regard to the proportion of estimated obese patient populations seen by the CDs (Table 1).

**Table 1:** Association of importance of teaching obesity related topics and percentage of overweight/obese patients seen in practice

% of patients OW &/or obese	Not at all important	Slightly important	Moderately important	Very Important	Extremely important	Total Percent
		n = 7	*n* = 28	*n* = 41	*n* = 18	
0–25% *n* = 3	(0%)	(0%)	(66.7%)	(0%)	(33.3%)	100%
26–50% *n* = 36	(0%)	(2.8%)	(33.3%)	(44.4%)	(19.4%)	100%
51–75% *n* = 48	(0%)	(12.5%)	(25.0%)	(43.8%)	(18.8%)	100%
76–100% n = 7	(0%)	(0%)	(28.6%)	(57.1%)	(14.3%)	100%

Fisher’s Exact test, *p* = 0.627

*OW* overweight

Almost 90% of the CDs responded that time was a barrier to teaching students about obesity. In comparison, knowledgeable faculty, resource limitations, and negative administration/faculty attitudes were each selected less than 5% of the respondents. The three most common methods of teaching obesity topics were case-based simulated patients, lectures, and on-line case based teaching (Table 2). The least common method was hands-on instruction. Table 3 lists the various obesity topics taught during clerkship. The most common topics taught were co-morbid conditions associated with obesity, diet, and exercise for weight loss. The least common topics taught were cultural aspects of obesity, obesity bias, medications that can cause weight gain, medications to treat obesity, and bariatric surgery. Participants were also asked whether or not they planned changes to the obesity curriculum over the next three years (Table 4). The majority of CDs are planning no changes to the existing curriculum.

**Table 2:** Teaching methods (*n* = 78)

Method	%
Didactic lecture	44.9%
Case based with a physician	51.3%
On-line case based	42.3%
Hands on (e.g., cooking class)	6.4%
Having written objectives about obesity	24.4%
Simulated patient with obesity	17.9%

Respondents were allowed to make multiple choices, so the sum of the numerators is > 78

**Table 3:** Obesity topics (n = 78)

Topic	%
Cultural aspects of obesity	29.5%
Comorbid conditions associated with obesity	82.1%
Obesity bias	12.8%
Diet for weight loss	76.9%
Exercise for weight loss	76.9%
Medications that can cause weight gain	26.9%
Medications to treat obesity	26.9%
Bariatric surgery	17.9%

Respondents were allowed to make multiple choices, so the sum of the numerators is > 100%

**Table 4:** Number of topics related to attitude

	SI	MI	VI	EI	*P*-value
	n = 7	n = 28	*n* = 42	n = 18	
Number of obesity related topics taught (Overall)^a^	0.9 ± 1.2	2.1 ± 1.8	3.3 ± 2.0	3.6 ± 1.9	*p* < 0.001
Number of advanced topics taught^b, c^	0.1 ± 0.4	0.5 ± 0.9	1.0 ± 1.5	1.4 ± 1.3	*p* = 0.019

*SI* Slightly important, *MI* Moderately important, *VI* Very important, *EI* Extremely important

^a^EI vs. SI, *p* = 0.003; VI vs. SI, *p* = 0.002; VI vs. MI, *p* = 0.028, all other comparisons were *p* > 0.05

^b^EI vs. SI, *p* = 0.043; EI vs. MI, *p* = 0.043, all other comparisons were *p* > 0.05

^c^Advanced topics include cultural aspects of obesity, obesity bias, medications than can cause weight gain, medications to treat obesity, and bariatric surgery

Table 5 shows results for comparisons of levels of importance of teaching obesity related topics during clerkship with regard to number of topics taught. The more important a CD thought teaching obesity topics was, the more topics that were covered. Specifically, there were significant differences in CDs who selected EI compared to selecting SI, those who selected VI compared to SI and those who selected VI compared to MI. Results for comparisons of advanced topics covered (cultural aspects of obesity, obesity bias, medications than can cause weight gain, medications to treat obesity, bariatric surgery) showed significant differences between EI and SI, as well as between EI and MI.

**Table 5:** Multiple regression analyses of number of obesity topics taught

Dependent variable	Independent variable	b-coefficient	*p*-value
		(95% CI)	
Overall number of obesity topics taught^a^	Clerkship duration^b^	0.29	0.072
		(−0.03–0.61)	
	Attitudes:	0.64	< 0.001
	VI vs. SI/MI^c^	(0.30–0.99)	
	Attitudes:	0.77	0.001
	EI vs. SI/MI^c^	(0.33–1.21)	
Number of advanced topics^d^	Clerkship duration^c^	0.12	0.430
		(−0.18–0.42)	
	Attitudes:	0.29	0.074
	VI vs. SI/MI^c^	(−0.03–0.62)	
	Attitudes:	0.65	0.002
	EI vs. SI/MI^c^	(0.24–1.06)	

EI: Teaching obesity related topics was extremely important; VI: Teaching obesity related topics was very important; MI: Teaching obesity related topics was moderately important; SI: Teaching obesity related topics was slightly important

^a^Total number of obesity topics taught during the clerkship

^b^Clerkship duration was either < 6 weeks or > 6 weeks (< 6 weeks was the reference variable)

^c^Attitudes compared either VI or EI vs. the reference variable, SI or MI

^d^Less commonly discussed topics (any study topic taught by < 30% of the study sites). The included topics were cultural aspects of obesity, obesity bias, medications that can cause weight gain,medications to treat obesity, and bariatric surgery

Results of the regression analyses are shown in Table 6. There was no significant association between clerkship duration (< 6 weeks, > 6 weeks) and number of topics taught or advanced topics taught. There were significant associations with regard to number of topics taught and importance level of obesity education during clerkship. With regard to all topics taught, programs with CDs who selected VI had 0.6 more obesity topics taught than programs with CDs who selected SI/MI and programs with CDs who selected EI had 0.8 more obesity topics taught than programs with CDs who responded SI/MI. When looking at advanced topics only, programs with CDs who chose EI had 0.6 more topics taught than programs with CDs who chose SI/MI.

## Discussion

To our knowledge, this is the first survey among family medicine CDs about curriculum regarding obesity. The study showed that the most common barrier to teaching about obesity during family medicine or primary care clerkships was time constraints (89%). However, clerkships that were relatively longer than others (> 6 weeks) did not have any significant relationship with the number of obesity topics taught. The CDs who placed more importance on obesity education, on average, taught more obesity topics (both in terms of overall number and number of advanced topics) than those who viewed it less importantly. Programs with CDs who viewed obesity education as extremely important taught 0.8 more topics than CDs who viewed obesity education as somewhat or moderately important. This could represent advanced topics, such as obesity bias or bariatric surgery.

It was expected that CDs would place greater importance on obesity education if their patient population had a higher percentage of obese patients, although this was not the case in our study. The majority of clerkship directors identified their obese patient population to range from 26 to 75%, which is consistent with the US population. Seven CDs reported a patient population with > 75% obesity. However, this number could represent obesity fellowship trained physicians, or geographic location of the CDs.

Despite more than one third of the US population being affected by obesity, this survey showed family medicine clerkships are not consistently including many obesity topics other than diet and exercise, and co-morbid conditions associated with obesity. Only 10% of CDs endorsed teaching about obesity bias, the need for which is shown by a 2013 study that revealed nearly 40% of medical students surveyed had an anti-fat bias, with 67% of that population being unaware of it [14]. Another study surveying medical students reported obesity as the most common cause for derogatory remarks to be made in reference to a patient [15]. This problem is not unique to the US, as a United Kingdom survey of healthcare students training to become physicians, nurses, nutritionists, or dieticians, showed that students had an overall average negative attitude towards the obese population, with 10.5% having high levels of fat phobia [16]. Additionally, more than half of the CDs are planning no changes to existing curriculum in the next three years regarding obesity, while obesity prevalence is expected to increase 33% from 2010 to 2030 [17].

There are several limitations to this study. Approximately 30% of those surveyed did not respond and not all of those who did respond answered every question. Differences between those who did respond and those who did not respond were not assessed. When asked about the obese patient population, the answers were based on the CDs’ estimate, rather than an actual determination of the percentage of obese patients in their practice. Moreover, a CD’s own obesity bias may affect the education provided on this topic during clerkship influencing responses to survey questions and the estimation of their obesity patient population. Further, the number of topics covered during the clerkship does not equate to the depth or quality of teaching of the topic. Time spent on topics taught and curriculum for the topics was not evaluated in this study. This goal of the survey was to evaluate the emphasis placed on obesity education, not the efficacy of such education.

Future research should examinethe effectiveness of obesity education on medical students approach to treating patients with obesity. Will students who have more obesity education be better able to understand and change their patients nutritional habits?. As organizations develop their obesity curriculum, the various advanced topics, such as obesity bias, need to be considered and assessed for possible introduction to the mainstream curriculum. Future studies should also assess involvement of obesity fellowship trained teaching faculty who can lead/conduct large scale clinical trial in order to achieve a clinically meaningful weight loss for obese patients.

## Conclusions

We found there are some topics, such as co-morbid conditions associated with obesity, diet for weight loss, and exercise for weight loss, that are taught (> 75%) during family medicine clerkships. Other topics are much less commonly taught, including obesity bias, medications related to obesity, and cultural issues around obesity. The key finding in this study is that the most commonly rated barrier to teaching about obesity during the family medicine clerkship is time constraints, yet obesity content in the clerkship is more correlated with CD attitude. If CDs felt more strongly about obesity education, they taught a higher number of core topics, as well as a higher number of advanced topics despite the length of the clerkship.

We hypothesized most family medicine CDs will plan to increase the curriculum in the next three years but it appears more than half of them will plan no changes to their existing curriculum. Our results show that lengthening a family medicine clerkship will not necessarily improve the amount of obesity education, but CD attitude was shown to affect the number of obesity related topics covered. This suggests the potential need for CDs and others involved in family medicine education to assess their own biases regarding obesity. In so doing, we may more strongly confront this nationwide epidemic by better preparing the doctors of the future.

## Abbreviations

CACMS: Committee on accreditation of canadian medical schools
CAD: coronary artery disease
CAFM: Council of academic family medicine’s
CD: clerkship director
CDC: Center for disease control
CERA: Educational research alliance
DM: diabetes mellitus
EI: and extremely important
HTN: hypertension
LCME: Liaison committee on medical education
MI: moderately important
NCHS: National center for health statistics
SI: slightly important
